# Antibodies in tuberculosis: functional capacity as key determinant

**DOI:** 10.1128/iai.00394-25

**Published:** 2026-01-21

**Authors:** Krista E. van Meijgaarden, Patricia S. Grace, Natalia T. Freund, Jacqueline M. Achkar, Thomas Lindenstrøm, Joshua Tan, John Chan, Carolyn G. King, Tom H. M. Ottenhoff, Simone A. Joosten, on behalf of CTVD B cell group

**Affiliations:** 1 Leiden University Center for Infectious Diseases (LUCID), Leiden University Medical Center 4501, Leiden, The Netherlands; 2 Department of Microbiology and Molecular Genetics, University of Pittsburgh School of Medicine 12317 , Pittsburgh, Pennsylvania, USA; 3 Department of Clinical Microbiology and Immunology, Gray Faculty of Medical and Health Sciences, Tel Aviv University 26745, Tel Aviv, Israel; 4 Department of Microbiology and Immunology, Albert Einstein College of Medicine 2006, Bronx, New York, USA; 5 Department of Infectious Disease Immunology, Center for Vaccine Research, Statens Serum Institut 4326, Copenhagen, Denmark; 6 Antibody Biology Unit, Laboratory of Immunogenetics, National Institute of Allergy and Infectious Diseases (NIAID), National Institutes of Health (NIH) 2511, Rockville, Maryland, USA; 7 Department of Medicine, Center for Emerging Pathogens, Public Health Research Institute, New Jersey Medical School, Rutgers University 214907, Newark, New Jersey, USA; 8 Department of Biomedicine, Infection Immunology Laboratory, University of Basel 27209, Basel, Switzerland; University of California Merced, Merced, California, USA

**Keywords:** functional assays, antibodies, tuberculosis

## Abstract

The human immune system employs both innate and adaptive mechanisms to control pathogens, with antibodies playing a pivotal role in immune memory and defense, in particular against viral infections. In tuberculosis, antibody titers have long been used to assess immune responses, but their presence alone fails to predict protective efficacy. Recent studies highlight that antibody functionality is critical for effective immune activity. Despite widespread detection of *Mycobacterium tuberculosis* (Mtb)-reactive antibodies in individuals with active disease, *Mtb* infection, and even in healthy controls, their potential to control *Mtb* growth is variable and only detected in a proportion of individuals. This perspective emphasizes the need for robust functional assessment of antibodies to better understand their role in mycobacterial control and inform vaccine development. Notably, antibodies binding to purified protein derivative of *Mtb*, a mixture of degraded antigens from *Mtb* cultures, are widespread but not universally functional, underscoring the importance of Fc characteristics and epitope specificity. Initial high-throughput screening using phagocytosis and direct mycobacterial binding assays is an active indicator of antibody function. By refining and combining existing assays, as recommended in this perspective, we can better characterize antibody contributions, particularly their immunomodulatory potential, toward improved control of *Mtb*. Albeit antibodies may not be essential in natural protection, functional antibodies induced by vaccination may be of added value and contribute to host protection.

## INTRODUCTION

## PART 1: ANTIBODIES AND B CELLS IN TUBERCULOSIS

The human immune system is complex, but intriguing, with both the innate and adaptive arms acting synergistically to achieve pathogen control and build immunological memory at the same time. Antibodies are a key component of the memory response, produced by B cells in a primary response. They can recognize, bind, and neutralize pathogens. Antibody specificity, affinity, and avidity are essential for effective antibody-mediated pathogen control and can be increased through repeated exposure to the antigen, either by infection or vaccination. Antigen-binding by the fragment antigen-binding (Fab) domain is increased by somatic hypermutation and affinity maturation, improving recognition of the pathogen and its antigens. Depending on the inflammatory environment, in chronic or persistent infections, antibodies undergo post-translational modifications, like glycosylation, that can affect their ability to mediate various immune functions. However, for pathogens that preferentially dwell within host cells, the possible contribution of antibodies to their control is less obvious and often debated but may be significant (1–4).

The disease tuberculosis (TB), caused by *Mycobacterium tuberculosis (Mtb*), is globally still the deadliest infectious disease caused by a single pathogen (5). While *Mtb* infection can progress to disease in the first months to years of exposure, the differences in disease progression may, in part, be due to the bacteria’s ability to evade the immune system, surviving in a dormant state in macrophages. As an intracellular pathogen, the main defense of the immune system was previously considered to be mediated by adaptive T-cell immunity which, due to its relatively delayed induction, primarily works to prevent bacterial dissemination. More recently, interest in B-cell responses and, in particular, the role of antibodies in TB pathogenesis has been emerging with accumulating evidence for their role in controlling infection (6–10). The contribution of B cells has been studied in various murine TB models, including B-cell-deficient mice, with results depending on both the route of infection and the timing of B-cell ablation. In models using aerosol infection, comparable bacterial loads were seen in lungs of wild-type (WT) and B-cell-deficient mice (11), whereas B-cell-deficient mice infected intravenously with *Mtb* had higher bacterial burdens than WT mice (12). Importantly, despite similar bacterial loads in lungs of B-cell-deficient and WT mice, WT mice had fewer pulmonary granulomas and delayed dissemination of *Mtb* from the lung to other organs. B-cell-deficient mice resembled WT mice when reconstituted with naïve B cells (13). Here, lesion development and bacterial control were uncoupled, with lung pathology, but not bacterial load, being critical for survival, highlighting a role for B cells and altered cellular infiltrates in shaping the inflammatory response during Mtb infection. Furthermore, in Rhesus macaques aerosol-vaccinated with recombinant *Mtb*, B-cell depletion, rather than vaccination, led to significantly increased bacterial burden in lung sections and individual granulomas, along with reduced accumulation of T_FH_-like cells during high-dose *Mtb* CDC1551 infection (14). Though the impact on bacterial burden is variable across all models, it is clear that B cells play an important role in reducing pathology during infection. In human studies, circulating dysfunctional B cells, with impaired proliferation, antibody and cytokine production, were observed in individuals with TB disease, but upon treatment, this was restored (15). Increased memory B-cell phenotypes were noted in patients with TB at the time of diagnosis compared to end of treatment and following BCG vaccination (16, 17). Compared to other lung diseases, lower levels of marginal zone and mature B cells, increased levels of activated B cells, switched B cells, and plasma cells were detected in individuals with TB disease at time of diagnosis, pointing to a contribution of B cells during active disease (16). Lung tissue from patients with TB has been described to contain multiple B-cell populations, typically within granuloma-associated tertiary lymphoid structures. These include tissue resident memory, germinal center, antibody-secreting, proinflammatory atypical as well as regulatory B cells, with several being expanded in TB disease (18). Taken together, these studies indicate that B-cell phenotypes are altered in TB disease, and as shown in mice, perhaps contribute to immune modulation in infected humans.

### Antibodies against *MTB*

*Mtb* is composed of a complex cell wall and the bacterium contains close to 4,000 proteins. The diversity of antigens recognized is large and includes proteins, glycolipids, lipoproteins, and secreted proteins (7, 19–22). Antibodies binding many of these *Mtb* antigens are detected in individuals with TB disease, *Mtb* infection, healthy household contacts, and even healthy controls. However, it has been challenging to link antigen-specific titers to protection, infection, or risk of TB progression because the antigen-specific repertoire is so heterogeneous (19–25). A well-studied TB antibody target of polyclonal antibodies is the immunogenic mycobacterial surface glycan (lipo) arabinomannan ((L)AM), detected at various stages of TB disease or infection. These AM-specific antibodies increased the growth control of intracellular *Mtb* in THP-1 monocytes, and with passive transfer reduced bacterial burden in lungs of *Mtb*-infected mice (10). BCG vaccination increased AM-specific IgG titers in humans, and post-vaccination sera enhanced phagocytic capacity and phagolysosome fusion compared with pre-vaccination sera (26). Vaccination of mice with an AM-Ag85B conjugate induced high antigen-specific titers in mice, and these antibodies, when passively transferred to naïve mice, decreased bacterial burden in lung and spleen of immunized and *Mtb*-infected mice compared with those immunized with single antigen (27). Broad antibody profiling in individuals with TB disease or infection demonstrated that the HIV infection status modulates antibody recognition of *Mtb* antigens (28). Additionally, *Mtb* infection and active TB disease could be discriminated based on both antibody titres and Fc receptor (FcR)-binding characteristics. Strikingly, more than 70% of 209 tested antigens were recognized by antibodies, either subclass or isotype-specific or less restricted (28). Collectively, while the data point to some antigenic targets contributing to antibody-mediated bacterial control, we still do not know and understand what the critical antigenic targets are.

Antibody titers against *Mtb*-specific antigens fail to discriminate states along the spectrum of *Mtb* infection (24, 25, 29–34). In more recent antibody screening approaches that focus on the isotype, subclass, and glycosylation of the antibody Fc domain, antibody Fc features were able to discriminate *Mtb*-infected individuals from patients with TB disease for purified protein derivative (PPD)-binding and Ag85A/B-specific IgM antibodies (35). In non-human primates, IgG, IgA, and IgM titers in plasma, but especially in BAL fluid 4–8 weeks after vaccination, were highest in those vaccinated intravenously, compared with intradermal or aerosol routes with high levels of protection in the intravenous BCG group (36, 37). Upon *Mtb* challenge, higher antigen-specific IgM titers were associated with reduced bacterial burden in lungs at necropsy. Independent aerosol vaccination experiments with an attenuated *Mtb* strain showed increased IgM titers and not IgG nor IgA, in attenuated-*Mtb* vaccinated animals with associated reduced bacterial burden, indicating IgM as a contributor to protective immune responses (36). Furthermore, pulmonary but not intradermal BCG vaccination prevented infection in a repeated low dose *Mtb* challenge study in non-human primates, with increased IgA levels in BAL fluid as one of the correlates of local protective immunity (38). Higher titers of AM-specific IgA in the lungs of cynomolgus macaques prior to *Mtb* infection were associated with the ability of the animals to control the infection, supporting a role for different isotypes at varying states of *Mtb* infection, in particular at the mucosal surface (39). In addition, human TB lungs contain high levels of *Mtb*-reactive antibodies with the capacity to promote *Mtb* phagocytosis (18).

### Antibody-Fc interactions

In humans, IgG antibodies have been assessed more extensively. While IgG3 normally is a less abundant IgG isotype, these *Mtb*-antigen-specific titers were even decreased in individuals with recurrent TB disease, compared with those without recurrences (40). In line with the decreased IgG3, complement factors C4 and C3, known to be activated by IgG3, were found to be lower in 23% of individuals with a confirmed relapse out of 130 individuals with TB disease (41). Increased levels of PPD-binding IgG4 were detected in individuals with TB disease compared to individuals with *Mtb* infection or treated for TB disease, suggesting poorly functional antibodies in those with active disease (35). IgG4 depletion enhanced antibody-dependent neutrophil phagocytosis (ADNP) and natural killer (NK) cell activation (ADNKA) by PPD-binding antibodies (35). Lu et al. demonstrated Fc-mediated functional effects for *Mtb*-specific antibodies. Antibodies from *Mtb*-infected individuals enhanced macrophage activation and *Mtb* growth restriction compared with antibodies from individuals with TB disease, without differences in antigen-specific titers between both groups (6). To further investigate the significance of the isotype, monoclonal antibodies were Fc-engineered and functional properties evaluated. In C57B6 mice, exchange of the human Fc-IgG1 domain from a monoclonal antibody (mAb) against LAM for the less functional mouse IgG1, functional mouse IgG2a, or mouse Fc-IgG2a point mutant led to loss of FcR-binding, and FcR affinity and cellular engagement were affected (42). In line with these data, the high affinity human mAb P1AM25, targeting multiple LAM/AM epitopes, showed *in vivo* protection when given as murine IgG2a but not IgG1 or the non-FcR-binding IgG1-D265A mutant to C57B6 mice prior to *Mtb* infection (7). Furthermore, Fc-engineered antibodies derived from a human monoclonal α-glucan-specific IgG1 demonstrated FcR-dependent characteristics, determined by point mutations in the Fc domain, affecting receptor binding, phagocytic capacity by monocytes or neutrophils, and NK cell activation (43). Building on these findings, the capacity to restrict *Mtb* growth by the Fc variants was studied in whole blood assays, assessing the relationships with the functional assays. Here, *Mtb* killing correlated with neutrophil-dependent phagocytosis (43), likely due to the high number of neutrophils in a whole blood-based assay. Similar importance of FcR interactions in functional responses was also demonstrated in other complex infectious diseases like malaria, where the engineering of Fc domains of mAbs resulted in enhanced binding to FcRs with a corresponding, though modest, effect (44). Moreover, antibody function may depend on the environment, including the presence of less functional antibodies targeting the same antigen. IgG4 antibodies for instance, which are known to bind poorly to NK cell FcγRIII (CD16), have been shown to inhibit ADCC activity of other IgG subclass antibodies by competing for the same antigenic sites (45). Taken together, these data show that antibody-FcR interactions are essential functional characteristics besides and likely independent of antigen specificity.

Antibodies may contribute to reduced bacterial loads *in vivo*, as has been demonstrated in several passive transfer studies (7–9, 36). Polyclonal antibodies directed against many different recombinantly expressed *Mtb* proteins, isolated from *Mtb*-exposed, but not infected, health care workers, protected mice against subsequent *Mtb* challenge (8). Similarly, PstS1-specific antibodies reduced BCG and *Mtb* levels in whole blood growth inhibition assays; an effect that could be abolished by an aglycosylated Fc variant with no binding to FcγRs and blocked by FcR blocking antibodies (9). *In vivo,* these same antibodies prophylactically reduced colony-forming units (CFUs) in lungs of *Mtb-*infected mice in agreement with the growth inhibition results. Similarly, passive transfer experiments with a high-affinity human mAb against AM provided protection in *Mtb* infected C57B6 as well as FcγR-humanized mice by increasing *Mtb* phagocytosis and reducing intracellular bacterial growth in macrophages (7). Collectively, these data suggest that antibodies may have the capacity to contribute to *Mtb* control, but not necessarily all antibodies do have that capacity. Antibody specificity so far cannot be correlated to functional properties, a field that deserves more attention. Intriguingly, when antibody specificity remained constant and Fc domain properties were varied, abundant differences were identified in the capacity to control mycobacteria, suggesting dominance from the Fc domain in functional capacities of antibodies (42).

Evaluation of antibodies should thus be extended beyond measurement of only titers to include also functional capacities. Once functional antibodies have been identified, these can be assessed in great detail to unravel the properties that drive their activity.

## PART 2: PERSPECTIVE ON FUNCTIONAL ANTIBODY MEASUREMENTS IN TB

### The need to measure better, not just more

**Fig 1:**
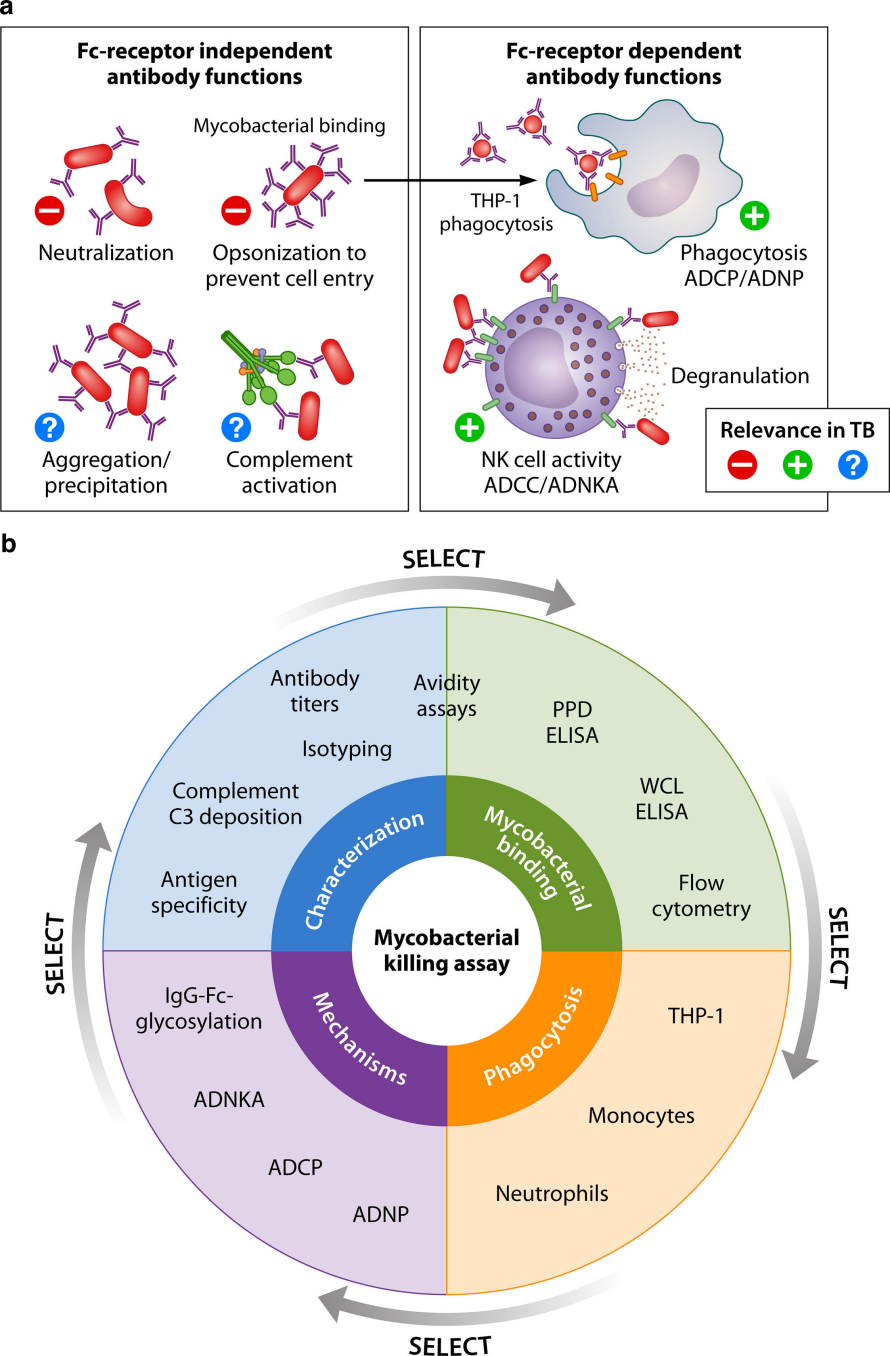
Antibody testing in relation to the functional capacity for control of mycobacterial growth. (**a**) Representing all Fc-independent and dependent antibody functions with relevance in relation to TB. The left panel shows all Fc receptor-independent antibody functions for which there is limited to no evidence that they can directly influence the infection with Mtb or TB disease. The right panel shows the antibody contribution in Mtb infection through their Fc domain in phagocytosis and NK cell activities. ADCC, antibody-dependent cellular cytotoxicity; ADCP, antibody-dependent cellular phagocytosis; ADNKA, antibody-dependent NK cell activation; ADNP, antibody-dependent neutrophil phagocytosis. (**b**) Schematic wheel demonstrating all assays currently used in the field recognizing the different characterization steps leading to the functional contribution of antibodies to mycobacterial growth control. WCL, whole-cell lysate. Figure created in BioRender.com.

The importance of antibody function rather than antibody titres is not unique for TB, but seems more generalizable for protection against infectious agents. More specifically, modulations of the immune systems, such as by metabolic perturbations or other comorbidities, may affect function more dramatically as compared to titers. For example, antibody titers elicited by influenza or SARS-CoV-2 vaccinations were largely similar between healthy individuals, obese individuals, or individuals with type 2 diabetes, but the quality of the antibody response was strongly influenced not only by age and genetic factors but also by these underlying comorbidities (46). Furthermore, while IgG titers for the SARS-CoV-2 spike protein did not change over time in a prospective household cohort study, the IgG avidity was significantly increased ten months after first measurements (47). Individuals exposed to *Mtb* but negative for both the tuberculin skin test (TST) and interferon gamma release assay (IGRA) exhibited higher IgG avidity for PPD compared to TST/IGRA-positive individuals, suggesting prolonged antigen exposure and enhanced affinity maturation. This finding may seem counterintuitive, as IGRA positivity reflects robust T-cell activation. However, IGRA-positive individuals typically mount a strong Th1-driven cell-mediated response, which can limit extensive humoral development. In contrast, IGRA-negative exposed individuals may have experienced low-level or persistent antigen exposure without dominant Th1 activation, allowing more sustained B cell-Tfh interactions and progressive affinity maturation (48, 49). Furthermore, increased avidity was also shown in sera from non-human primates vaccinated with BCG intravenously compared with intradermal or aerosol vaccinated animals, accentuating that besides natural exposure, vaccination can improve the quality of immune responses (50). Thus, antibody titers by themselves are not directly correlated with effective immune responses. The presence of antibodies is insufficient to address critical questions related to correlates of disease or protection, immune modulation, reduction of pathology, and mycobacterial killing, all of which are central to TB research (51). While accumulating data demonstrates the involvement of antibodies against TB, we need to improve our understanding of the contribution of antibodies during various stages of infection and disease and what they contribute functionally, including the impact of antibodies at the site of infection: lungs, airways, and oral mucosa. Here, we illustrate the possible contributions of antibodies in the context of *Mtb* infection and TB disease (Fig. 1a) and outline functional antibody characterization assays. From screening for mycobacterial binding and phagocytosis to mycobacterial growth restriction while acknowledging the importance of antibody characterization, such as subclasses, isotypes, antigen specificity, and posttranslational modifications (Fig. 1b).

### Phagocytosis and cellular assays for opsonized bacteria—the pros and cons

Antibody functional capacities can be determined by various *in vivo* and *in vitro* assays. *In vivo* experiments are best suited for testing mAbs, as they require larger quantities of specific antibodies. In contrast, evaluating numerous polyclonal IgG samples in animals would pose an ethical burden, offer limited insight into defined antibody epitopes and Fc characteristics, and be both labor-intensive and costly. For these reasons, *in vitro* assays are often preferred as a practical and efficient approach for the initial qualification of polyclonal antibody responses against mycobacteria.

With the thick and complex, lipid-rich cell wall of *Mtb* as a physical barrier for direct lysis initiated by antibody binding, the effects of antibody binding may likely involve immune activation rather than a direct antibacterial result. Complement and Fc receptors, Toll-like receptors, nucleotide-binding oligomerization domain-like receptors, scavenger receptors, and mannan-binding lectin can all facilitate the access of *Mtb* into host cells (52). However, this entry is two-sided, as *Mtb* on one hand can only survive intracellularly by arresting phago-lysosomal fusion in macrophages, advantageous for the bacterium; on the other hand, once intracellular, *Mtb* is a target for T-cell-mediated immune responses, which can benefit the host by triggering cytotoxic effects on infected macrophages that enhance their ability to kill pathogens.

Opsonization assays for *Mtb* or BCG and subsequent phagocytosis are valuable starting points for studying the functional contribution of antibodies, even when they do not fully address the questions related to *Mtb* killing and bacterial burden (6, 53, 54). They can easily be performed at high throughput for larger sample numbers using bacterial cultures and a phagocytic cell line as a first step to select interesting samples for further functional analysis. Most antibody-dependent cellular phagocytosis (ADCP) assays use a monocytic cell line, frequently THP-1, to assess the capacity of different sera or mAbs to enhance phagocytosis of antigen-coated beads, BCG, or H37Ra. The virulent lab strain of *Mtb*, H37Rv, is not often used in this context as this requires higher safety level laboratory facilities. Here, BCG or the attenuated H37Ra strain can serve as suitable surrogates as the mycobacterial capsule—including LAMs—are highly similar (if cultured in the right conditions), whereas most virulence factors that differ between the strains are not expressed on the surface but rather secreted and thus likely not critical for phagocytosis (55). Mycobacterial culture conditions, including the use of detergents, can alter antigen exposure on the bacterial surface, which in turn affects antibody binding and may influence the outcome of functional assays (56).

In addition to promoting phagocytosis, antibodies may directly activate macrophages in FcR-dependent as well as FcR-independent manners. Antibody opsonized mycobacteria, either PstS1-specific monoclonals or polyclonal sera from IV BCG-vaccinated non-human primates, promoted NLRP3 inflammasome activation (57). Intriguingly, protection by passive transfer of non-human primate sera into infected mice was dependent on NLPR3 inflammasome activation, demonstrating functional relevance of antibody-induced inflammasome activation (57). Also, in humans, inflammasome activation was associated with antimicrobial activity, as in *Mtb*-infected individuals, IL-1β release (as indicator of inflammasome activity) was elevated compared to those with pulmonary TB (6, 48).

**Fig 2:**
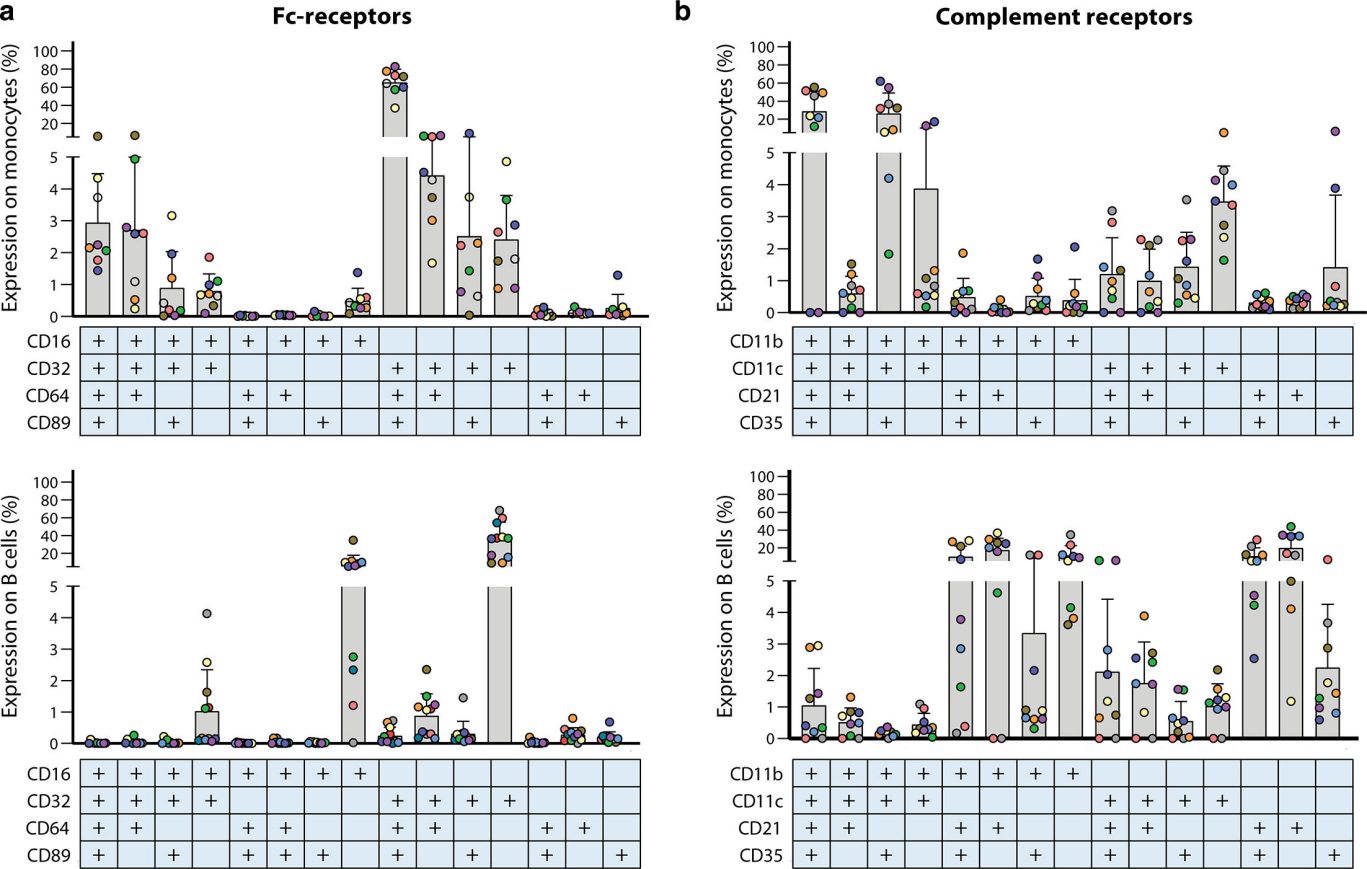
Fc- and complement-receptor surface expression profiles of monocytes and B cells are complementary and heterogeneous. PBMCs (*n* = 9) were analyzed by flow cytometry for the surface expression of (**a**) Fc receptors—FcyRI (CD64), FcγRII (CD32), FcγRIII (CD16), and FcαR (CD89)—and (**b**) complement receptors—CR3(CD11b), CR4(CD11c), CR2(CD21), and CR1(CD35) on monocytes and B cells. Boolean analysis was performed and expression on cell subsets is shown. Each color represents a donor and the tables below the *x*-axis represent the presence of a complement receptor. Data on Fc receptors (**a**) is re-used from van Meijgaarden et al. (53). Short methodology: Anonymous buffy coats from healthy blood bank donors were used upon consent for scientific use of blood products (Sanquin blood transfusion services, the Netherlands). PBMCs were isolated by Ficoll (Pharmacy LUMC, the Netherlands) density gradient separation. PBMCs were stained with vivid live dead stain (Invitrogen, ThermoFisher) and subsequently with CRs: CD11b-BV650 (clone D12), CD11c-PerCP-Cy5.5 (clone B-ly6), CD21-BV711 (clone B-ly4), CD35-BV510 (clone E11), or FcRs: CD16-PE-CF594 (clone 3G8), CD32-PE (clone 3D3), CD64-FITC (clone 10.1), CD89-APC (clone A59, BioLegend), and CD3-APC-Cy7 (clone SK7), CD19-BV605 (clone SJ25C1), CD56-PE-Cy7 (clone B159CD32-PE (clone 3D3), and CD14-BV786 (clone M5E2). Staining included Brilliant Violet Stain buffer (all BD Biosciences). Samples were acquired on a LSRFortessa (BD) and analyzed with FlowJo software v9.7 (Treestar Inc).

Phagocytosis by macrophages and neutrophils can be enhanced by the addition of complement, as FcRs and complement receptors (CRs) can function synergistically, through the recognition of opsonized pathogens. Although usage of phagocytic cell lines enables screening for large collections of sera, it does not necessarily cover the full potential of complement- and Fc receptor-mediated phagocytosis because not all receptors are present on these cell lines, e.g. THP-1 cells lack CR1 and FcγRIII (58). Primary neutrophils, peripheral mononuclear cells (PBMCs), isolated monocytes, whether or not differentiated into macrophages, would be more representative to assess phagocytic capacities given the natural expression and diversity of all existing FcRs and CRs, but these experiments are more cumbersome and the cell yields are limited. Monocytes and B cells co-express complementary combinations of FcRs and CRs, with donor-dependent variability in surface expression levels, underscoring the receptor diversity that may potentially influence immune effector functions in addition to variation at the antibody site (Fig. 2). While assay reproducibility is generally not an issue when using cell lines, the inter-individual variability among primary cell donors warrants careful consideration.

Phagocytosis by both monocytes and neutrophils is an important step in down-selecting sera for further functional assessment, but initial screening using monocytic cell lines might facilitate which sera to select. Monocyte phagocytosis can be performed with either human PBMCs or isolated monocytes from buffy coats, managing cell yields that can be used for reasonable numbers of sera. ADNP) assays using monoclonal or polyclonal purified antibodies can be performed in whole blood, as antigen-specific antibody concentrations can be adjusted to assess direct effects. However, this approach is not directly applicable to polyclonal sera, where antibody specificity and concentration cannot be precisely controlled. For comparative testing of sera, one would need purified neutrophils, which are fragile, have a short lifespan, and should be isolated quickly as neutrophils become activated easily, so gentle handling is recommended (59).

In addition to monocytes and neutrophils, antibody-mediated effector functions can be executed by NK cells. Antibody-dependent cellular cytotoxicity likely does not affect *Mtb* directly, but indirectly through killing of infected macrophages by ADNKA (6, 35, 60). ADNKA is limited to measuring NK cell activity and degranulation capacity for opsonized mycobacteria without evaluating bacterial burden. A challenge for these NK cell assays is the need for adequate numbers of isolated NK cells. NK cells, only 5–15% of PBMCs, require negative selection procedures to prevent activation, followed by measurements of degranulation by CD107a, cytotoxic molecules, and/or cytokines. Based on the lower level of throughput screening, these assays will likely be more effective downstream, when selections based on antibody binding and different phagocytosis assays have been completed. Alternatively, commercially available NK-92 cell lines can be transduced or transfected to express CD16 or whole blood assays could be developed to test sera using solely opsonized bacteria for both ADNKA and ADNP (45).

**Fig 3:**
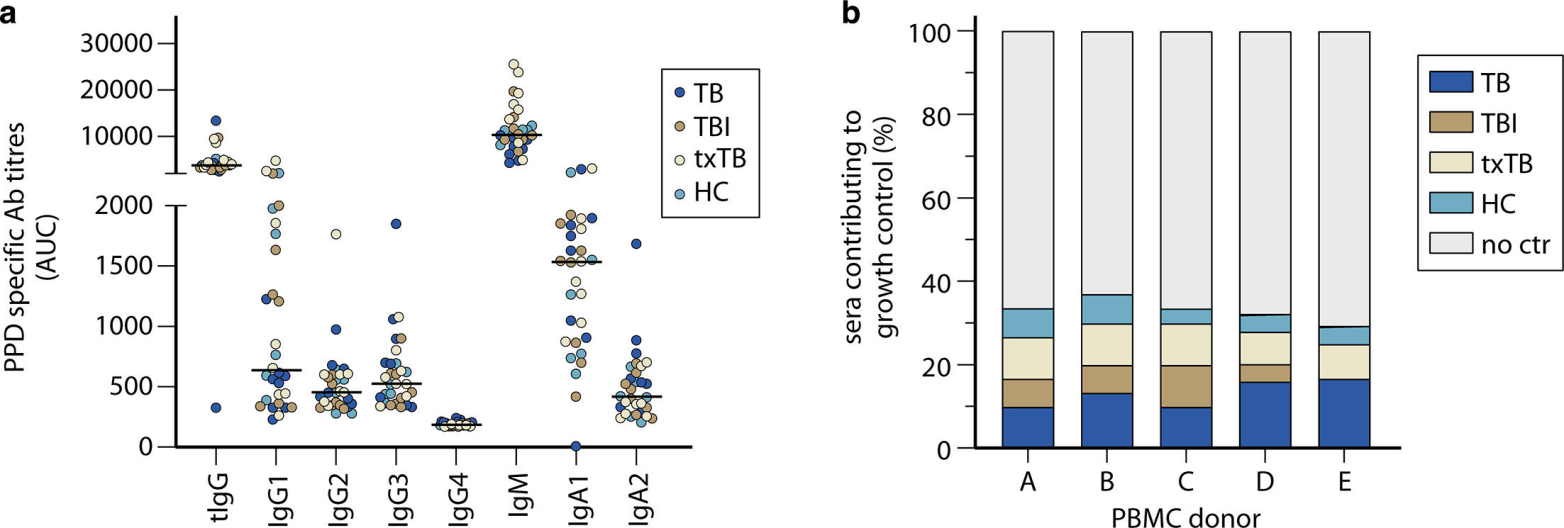
Antibody contribution to mycobacterial growth inhibition. Sera were collected from individuals with TB disease (TB) (*n* = 8), Mtb infection (TBI) (*n* = 8) or individuals after completion of treatment for TB (txTB) (*n* = 8). A healthy control (HC) group was added (*n* = 6). (**a**) Purified protein derivative (PPD)-specific titers for all 30 individuals were determined by testing sera in five dilutions (1:30, 1:100, 1:300, 1:1,000, and 1:3,000) to generate an area under the curve measurement using detection reagents for all Ig classes and subclasses and are plotted as AUC on the *y*-axis. Note, levels of antibody isotypes cannot be directly compared due to assay specifics. Between 24 and 30 sera were tested for their contribution to reduce BCG growth in five unrelated PBMC donors (A–E) in mycobacterial growth inhibition assays as previously described (53). (**b**) Growth control was defined by reduction of colony-forming units below the lower confidence limit. Coloring corresponds to disease grouping; individuals with TB disease in blue; Mtb-infected individuals in brown; individuals treated for TB in beige and healthy individuals in turquoise. Sera that did not contribute to growth control are shown in white. Data are re-used from van Meijgaarden et al. (53).

All assays described above highlight one aspect of the functional contribution of antibodies, but do not measure the overall blood-based capacity to inhibit mycobacterial growth, including unidentified cells, molecules, and mechanisms. Mycobacterial growth inhibition assay (MGIA) is an example of a robust *in vitro* assay that can be used to assess antibody effect on mycobacterial growth. MGIA has been employed to measure bacterial growth control between different human vaccination, disease cohorts, or animal models, including non-human primate vaccination studies (61–67). Recently, we adapted the MGIA to show that serum antibodies can contribute to CFU reduction (53). While we detected PPD-binding antibody levels in sera from all individuals with TB disease, individuals after completion of treatment for TB, individuals with *Mtb* infection and healthy controls (Fig. 3a). However, only 30% of these sera contained antibodies that could functionally contribute to mycobacterial growth control through Fc-mediated phagocytosis (53). This was irrespective of antibody titers or disease state (Fig. 3b), indicating again that TB-specific titers alone do not necessarily correlate with protective antibodies (53). Antigen-specific antibody titers and glycosylation profiles did not associate significantly with CFU reduction, suggesting that other factors had a crucial contribution (35, 53). In addition, the growth restriction was donor-dependent, partially due to donor PBMCs used as cellular source in this adapted MGIA, underlining the complex interaction between antibodies and immune cells for studying intracellular pathogens (53).

Independent of these functional assays, antibodies in sera of interest can be further classified from pan-antibody responses to in-depth analysis of subclass distribution, isotypes, antigen specificity, and posttranslational modifications. *Mtb* whole-cell lysates, comprised of surface (glyco- and lipo-) proteins, secreted proteins, and cell wall fractions, may serve as a potential first “screening-antigen” for antigen specificity. Ultimately, for specific research questions on (subunit-) vaccines, one may need to be more detailed on the antigens recognized; but neither cell lysates, PPD, nor single recombinant antigens can capture all antigens in their naturally occurring presentation including posttranslational modifications.

### Recommended assays for functional evaluation of antibody capacities

**Fig 4:**
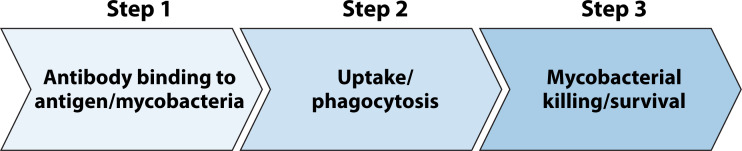
Flowchart to illustrate the selection of functional antibodies to ultimately measure mycobacterial survival. From antibodies binding to mycobacteria or antigens (step 1), to functional assessment by uptake or phagocytosis (step 2) to the measurement of the capacity to contribute to mycobacterial killing (step 3).

As a first step to select interesting serum samples for further functional analysis, high throughput analysis can easily be performed on large sample numbers using phagocytosis and direct mycobacterial binding assays (Fig. 4). Mycobacterial strains may provide variable results, but at this stage, no data are available that would be in favor of using H37Rv in the BSL3 environment above testing BCG or H37Ra in more accessible BSL2 laboratories, in particular for polyclonal responses as initial screening tools. Although related, readouts for bacterial binding as well as phagocytic potential are not measurements for the same function and may provide additive information. We therefore recommend selecting the best-performing sera for each assay and combining this with a subset of sera performing below average but not negative. Second, phagocytosis by monocytes, either within PBMCs or isolated, can be used to downscale the potential numbers of interesting sera. As inter-individual variability and relative abundance of specific cell types may influence heterogeneity of results, we propose to control for this biological variance by testing at least three independent monocyte donors for functional phagocytosis experiments and determine their FcR expression profiles as well as monocyte subset distribution (53). Again, top-performing and a subset of less functioning sera should be included to adequately measure the impact on bacterial growth in the subsequent step of bacterial killing assays, like MGIAs. MGIAs measure the sum of parts, immune cells, and serum factors, with functional control of mycobacterial growth as readout. Furthermore, supernatants can be taken after the coculture period for determination of soluble analytes like cytokines and chemokines as hallmarks of immune activation. Neutrophils, acting as “Trojan horses,” have the potential to activate the adaptive immune system and bridge innate and adaptive responses. Their phagocytic activity may offer valuable insights into mechanisms of mycobacterial control (68). However, this result should be analyzed separately as the functional MGIA to assess serum contribution is PBMC based.

Assays like the ones depicted in Fig. 1 can also be modified in order to gain further mechanistic insights, to investigate the relative contribution of specific pathways, but also to correlate phenotypic characteristics with functional properties. For example, Fc-mediated interactions can be specifically addressed in the analysis, using specific blocking reagents to dissect the contribution of specific FcRs and CRs. Besides, the involvement of complement can be measured by comparing serum with heat-inactivated serum, recognizing the importance of complement in boosting opsonophagocytic activity, antibody effector functions, and leukocyte recruitment, connecting important innate and adaptive immune responses in intracellular infections (69).

As discussed above, many individuals have PPD-binding antibodies, including healthy individuals without known exposure to *Mtb*, likely due to exposure to non-environmental mycobacteria, but not all antibodies are functional (6, 35, 53). Studies with (Fc-engineered) mAbs underline that antibodies likely need both epitope specificity combined with specific Fc characteristics to mediate a functional contribution, but investigating this knowledge gap for TB has just started (43). Furthermore, new TB vaccines currently in phases 2 and 3 development also induce antibodies; it would be highly interesting and relevant to study their relative contribution to control mycobacterial growth or mediate mycobacterial killing (70).

In summary, from this perspective, we have highlighted that antibody titers should be considered only a first step in the evaluation of humoral responses; they should be followed by the functional assessments of antibody-mediated effector responses. Functional assessment within groups that are clinically similar versus those with opposing outcomes may point toward important mechanistic insights, which can be employed to improve vaccines. We describe assays suitable for screening sera from human cohorts in a more functional manner that could potentially provide more mechanistic insights into the contributing roles of antibodies in the control of mycobacterial infections. Although performing these assays on large cohorts may seem ambitious, the proposed functional assays, being relatively inexpensive, high-throughput, and automatable without requiring high-end analyzers, make this goal achievable. The planned stepwise down-selection of samples for more labor-intensive assays, such as ADNKA and MGIA, should ensure representation of the entire study cohort. Furthermore, several of these assays can also be modified for the testing of mAbs. We anticipate that combinations of these assays will improve insights into the possible involvement of antibodies in TB. Hence, the main contribution of antibodies might well be their immune-modulating capacity, which will consequently be of added value to pursue in antibody-inducing vaccine strategies. The time has come to consider antibody functions as key contributors to better control of *Mtb*.
